# The Diagnostic Value of Examination of the Blood

**Published:** 1905-12

**Authors:** Walter A. Cowell

**Affiliations:** Olean, N. Y.


### The Diagnostic Value of Examination of the Blood.

By Walter A. Cowell, A. B., M. D., Olean, N. Y.

ARBITRARY therapy founded upon traditions and fables characterised medicine at its incipiency. Gradually, step by step, progress was made, and today the members of the medical profession are unanimous in the opinion that rational treatment depends upon accuracy of diagnosis, and the epigram of Pasteur "I do not know—I will investigate," states clearly our position before the world. Among the more recent and valuable aids which we have at our disposal is the examination of the blood.

The value of an exact and careful examination of the blood as a means of diagnosis is so well established that it needs no word of ours to fortify it in the position which has been accorded it by clinicians everywhere. It is not, by any means, an absolute method of making a diagnosis, but when taken in conjunction with physical and rational signs, it is very often a great aid and in some cases it is the determining factor without which our diagnosis must at best be only probable. Leube says, in his treatise of pernicious anemia, that only the results of careful examinations of the blood can give a firm support to the diagnosis, since none of its classical symptoms belong exclusively to that affliction. Again, when speaking of leukemia, he says: "In comparison to the results of the microscopical examination of the blood, all the other morbid phenomena of leukemia are of decided subordinate importance."

It is essentially fitting therefore to consider the value of the examination of the blood, and to briefly review a few of the more important facts of the blood pictures presented by the various affections of the blood-forming organs. The pallor of the patient, while suggestive of anemia, is not always a necessary accompanyment. As is well known, the color of the cheeks may depend upon the cutaneous capillary development of the individual. Thus, occasionally, are seen cases of pernicious anemia with red cheeks, and others which are pale in spite of the absence of anemia. The principle diagnostic facts to be learned in making the examination are:

(1) The number of erythrocytes (red blood cells) together with the sizes and shapes. (2) The number of the leukocytes (white blood cells) collectively, and the varieties present with their proportion. (3) The amount of hemoglobin. (4) The presence of organisms of a parasitic nature.

The technic of making the examination is so well known that I will not take the time in describing it except to say that the Türck plate is far superior to the older form, the Thoma-Zeiss plate, in as much as the field is nine times larger than the Thoma field and this greatly facilitates the white blood count, making it possible to make the count from one mounting.

In the study of the blood films I have found Wright's modification of Leishmann's stain to answer very well. It is suitable for office work because the films do not require the hardening and fixing which precedes the staining by Ehrlich's method. With it also the malarial parasites are readily seen.

The picture of primary pernicious anemia as distinguished from profound anemia of the cachexias is characteristic. The erythrocytes which in the adult male should number 5,000,000 to a cubic millimeter, are greatly reduced, reaching as low a figure in some cases as one half (½) or one quarter (¼) of a million to the cubic millimeter. The individual cases show great morphological changes, being drawn out in spindles and globular shapes. In this affection the hemoglobin loss is not as profound as that of the erythocytes and so the color index is a high one, after reaching 1.3, showing that the individual erythrocyte contains more than its usual amount of hemoglobin. We should therefore expect to find the erythocyte a deep red in the picture and this is what we do find. In contradistinction to this we find a less color index in chlorosis, since the main change is a loss of hemoglobin and here the individual erythocyte has a distinctive pale center.

In the picture we find large nucleated erythocytes or megaloblasts whose diameter is from 10-16 micro-millimeters. Ehrlich regards these as a distinctive finding in the blood of pernicious anemia. These cells are probably the predecessors of the non-nucleated erythrocytes whose diameter is the same as of the megaloblasts, and are very probably formed by the absorption of the nuclei of the megaloblasts. Laache claims that these are pathognomonic and they are a constant element of the picture. If, therefore, we should find megaloblasts and megalocytes in the blood picture in fairly large amounts and fairly constant, we have a case of pernicious anemia. The megaloblasts are only exceptionally met with in the blood of other anemias and are then very few. In conjunction with these cells, small nucleated and non-nucleated erythocytes having a diameter from 3.5-7 micro-millimeters, called microblasts and microcytes, are found.

Normal size nucleated erythrocytes are seen but are not significant of pernicious anemia. They simply show a great drain on the blood forming organs and have been called into service in the blood before complete maturity. They are also seen in the simple anemias as chlorosis. They are, however, of good prognostic import, showing that the blood-forming organs are functionating. In the profound or severest pernicious anemias these cells are exhausted, showing a lack of function of the bone-marrow and still less matured forms, the megaloblasts, mentioned above,, are carried into the blood. So we see that the greater the proportion of megaloblasts and megalocytes in a given case makes the diagnosis of pernicious anemia more certain.

The myelocytes, so characteristic of myelogenous leukemia, are sometimes seen in small numbers but never in the proportion obtained in leukemia. The individual erythrocyte in pernicious anemia has the property of taking more than one stain, making a mixed color, and has lost the property of forming rouleau. The leukocytes are as a rule diminished.

The type of pernicious anemia depending upon some primary affection and therefore called secondary pernicious anemia, is at times hard to differentiate. The affections found to produce such a condition are notably, syphilis, carcinoma of the stomach, chronic gastritis, severe and prolonged hemorrhages, continuous albumenous drain as from the nephritides and the typhoidal, malarial and cancerous cachexia in general. In such a secondary anemia the megaloblasts are absent, or if present are few. The erythrocytes are greatly reduced, numbering perhaps a million, and the hemaglobin is low, making a low index which may approximate the index of chlorosis. The individual erythrocyte is therefore pale in the center. There is slight leukocytosis mainly affecting the neutrophilic polynuclear variety. Nucleated cells, if present, are generally normal in size. The absence of megaloblasts in such a picture is proof of the secondary character of the anemia. The low index as opposed to the high index in pernicious anemia is very suggestive.

That there is some relation between a primary pernicious anemia and the subsequent development of carcinoma may be possible. Osler reports a case of primary anemia which was discharged as cured which returned in six years with a cancer of the stomach. In this connection I should like to call attention to a case which I had under observation and in which a carcinoma developed subsequently to primary pernicious anemia.

Mr. V. D., Rochester, Minn., age 54, clerk, born in U. S., married.

Complained of weakness and general debility. He saw physicians at his home who diagnosticated his trouble as cirrhosis of the liver and Bright's disease. His mother and one brother died of tuberculosis. No history of cancer or tumors in the family. Had diseases of childhood, also asthmatic attacks for years and frequent bilious attacks; heart good. Illness began in January, 1901. He began to get weak and at times was unable to attend to his work. A pale lemon color developed in his skin. No history of pain or chills or fevers. At night a puffiness was noticed above the shoe tops. Appetite variable. Tendency to diarrhoea. Lost breath upon slightest exertion. Weight not much affected but had lost strength. Came to New York in August, 1903. Examination at that time: Skin and mucous membrane pale and yellow. Pupil normal, pulse was large but soft. Thorax negative. Heart slightly enlarged and a haemic murmur heard over the pulmonary area, systolic in time. Abdomen was not tender nor was any tumor mass palpated. Patella tendon reflex normal as were other reflexes.

Urine contained slight amount of albumen. Upon examination of the blood the erythrocytes numbered 2,160,000 and the hemoglobin was 22%, making a color index of .6. But in spite of the low index the diagnosis of pernicious anemia of primary origin was made from the presence of megaloblasts and megalocytes. He was put upon treatment and left for his home much improved. In a few months, however, he gradually failed and died February 6, 1905. About 18 months after the patient went under treatment, upon autopsy made in Minnesota, besides the degeneration of the bone marrow and change in the viscera, a small, incipient carcinoma was found in the pancreas. I cannot say what variety of carcinoma it was, not having seen the specimen, but am relying upon the report of a pathologist.

From these two cases it would seem that there may be some connection between primary pernicious anemia and carcinoma.

Too much reliance should not be placed upon the color index since it is characteristic only in a general way. Leube says that exceptions to this rule occur in all forms of anemia and that some cases have a more marked diminution of hemoglobin than is in keeping with the number of erythrocytes. The presence of megaloblasts is the main basis for the diagnosis.

In chlorosis, the essential fact is a more pronounced diminution of the hemoglobin than of th erythrocytes and therefore a low color index. The hemoglobin may go down to 25%, while the erythrocytes may remain from 75-85%. In severe cases a few nomoblasts may be found and some change in the shape of the cells. The leukocytes are not influenced.

The coagubility of the blood is of interest in the affections so far considered. In the milder forms as chlorosis and the secondary anemia, there is a marked tendency for rapid coagulation with the formation of the thrombi in the extremities and cerebral sinuses, but in primary pernicious anemia the reverse is true and there is difficulty in checking slight bleedings. IN LYMPHATIC LEUKEMIA we find megaloblasts and nucleated erythrocytes, yet their presence is not distinctive. The principle feature is the predominance of the large and small lymphocytes, which may form as high as 95-98° of the white blood cells present. The myelocyte is absent in this form, probably the marrow not being as profoundly altered as in myelogenous leukameia.

IN ACUTE LYMPHATIC LEUKEMIA there is a predominence of the large lymphocytes and a diminution of the polynuclear varieties. The proportion of the erythrocytes and white cells may be as high as 1 to 1 instead of the normal 1 to 500.

IN CHRONIC LYMPHATIC LEUKEMIA the small forms of the lymphacytes are most conspicious and sometimes seem to be the only white blood cells to be found. Some of the large cells, so characteristic of acute lymphatic leukemia, may be found.

IN LEUCOYTE MYELOGENOUS LEUKEMIA there is a marked increase of the white cells, not only of the polynuclear neutrophilic variety, but also of the eosinophiles. The mast cells are increased and this is the only blood disease in which we get a marked increase of these cells. Their presence in increased proportion is therefore valuable from a diagnostic standpoint. The most characteristic finding, however, and upon which we base our diagnosis, is the presence in the circulating blood of eosinophilic and neutrophilic myelocytes. These show the profound change going on in the bone marrow. They may number 100, 000 in a cubic millimeter. Nomoblasts, and sometimes megaloblasts, are found. Megaloblasts have been seen in large numbers in myelogenous leukemia but the diagnosis is made principally from the mast cells and especially the myelocytes. The leukocytes in this picture are smaller than normally found and have been called "dwarf forms" of leukocytes.

Malaria is easily detected by finding the parasites in the blood corpuscles, and its variety can be told by the character of the development of the parasite.

I have briefly stated a few of the more important features of the blood attending the affections of the blood forming organs. The value of such findings can not be questioned in aiding the making of our diagnosis.

---

In a report on "The Non-operative Treatment of Hemorrhoids," read before the Elmira Academy of Medicine (*N. Y. State Journal of Medicine,* October, 1905), DR. ALFRED J. WESTLAKE says that the anusol suppository, composed of iodoresorcin-sulphonate of bismuth, zinc oxide and balsam peru with the excipient, will relieve pain better than any other in use today.

